# Severe bronchiectasis is associated with increased carotid intima-media thickness

**DOI:** 10.1186/s12872-024-04129-x

**Published:** 2024-08-28

**Authors:** Wang Chun Kwok, Kui Kai Lau, Kay Cheong Teo, Sze Him Isaac Leung, Chung Ki Tsui, Matthew S.S. Hsu, Kkts Pijarnvanit, Carman Nga-Man Cheung, Yick Hin Chow, James Chung Man Ho

**Affiliations:** 1 Department of Medicine, The University of Hong Kong, Queen Mary Hospital, 102 Pokfulam Road, Hong Kong, Pokfulam, Hong Kong SAR China; 2 State Key Laboratory of Brain and Cognitive Sciences, The University of Hong Kong, Pok Fu Lam, Hong Kong SAR China; 3 Department of Statistics, The Chinese University of Hong Kong, Shatin, New Territories, Hong Kong SAR China; 4 Department of Pathology, The University of Hong Kong, Queen Mary Hospital, Pokfulam, Hong Kong SAR China

**Keywords:** Bronchiectasis, Carotid initial thickness, Subclinical atherosclerosis, Cardiovascular disease

## Abstract

**Background:**

Although bronchiectasis has been shown to be associated with cardiovascular disease, there is limited evidence of an association with subclinical atherosclerosis, especially carotid intima-media thickness (CIMT).

**Methods:**

This prospective study compared CIMT among patients with and without bronchiectasis, and among bronchiectatic patients classified according to disease severity using the FACED score. The study was carried out at a major regional hospital and tertiary respiratory referral centre in Hong Kong.

**Results:**

Total 155 Chinese patients with non-cystic fibrosis (CF) bronchiectasis and 512 controls were recruited. The mean CIMT was 0.58 ± 0.10 mm, 0.63 ± 0.11 mm and 0.66 ± 0.08 mm respectively among controls, patients with mild-to-moderate bronchiectasis and patients with severe bronchiectasis. There was no statistically significant difference in CIMT between patients with mild-to-moderate bronchiectasis and controls. Multivariate linear regression revealed that CIMT was significantly increased in patients with severe bronchiectasis relative to controls. The same phenomenon was observed among patients without a history of cardiovascular disease or cardiovascular risk factors.

**Conclusions:**

CIMT was significantly increased in patients with severe bronchiectasis compared with controls without bronchiectasis, but not among patients with mild-to-moderate bronchiectasis, which suggested the subclinical atherosclerosis to be more prevalent among patients with severe bronchiectasis.

**Supplementary Information:**

The online version contains supplementary material available at 10.1186/s12872-024-04129-x.

## Introduction

Bronchiectasis is characterized by airway inflammation, abnormal mucus clearance and bacterial colonization with consequent progressive airway destruction and distortion. There is accumulating evidence that airway inflammation and immune dysregulation play a central role in the evolution of non-CF bronchiectasis [1].

An association of systemic inflammation with cardiovascular diseases has been demonstrated, while baseline C-reactive protein (CRP) level has been shown to predict the long-term risk of a first myocardial infarction, ischemic stroke, and peripheral artery disease [2–4]. Guidelines suggest measurement of high-sensitivity CRP in patients at intermediate risk of coronary heart disease (CHD) [5–7]. Other inflammatory markers such as interleukin-6, [8, 9] leukocyte enzyme myeloperoxidase, [7, 10–13] white blood cell count, erythrocyte sedimentation rate, IL-18, tumor necrosis factor alpha, transforming growth factor beta, soluble intercellular adhesion molecule-1, P-selectin, cathepsin S, and lipoprotein-associated phospholipase A2 have also been reported as markers of increased CHD risk [12, 14–20].

There is growing evidence of an association between bronchiectasis and cardiovascular diseases [21–26]. Bronchiectasis was associated with the development of cardiovascular disease in a population-based study conducted in the United Kingdom, as well as increased risks for coronary heart disease and stroke [22].

To assess underlying atherosclerotic burden and predict adverse cardiovascular events, various non-invasive functional and structural surrogate markers of vascular health can be measured. These include assessment of endothelial function by brachial-artery flow-mediated dilatation (FMD), and measurement of carotid intima-media thickness (CIMT) and arterial stiffness and ankle-brachial index (ABI). A case control study also showed that patients with bronchiectasis were at greater risk of endothelial dysfunction as measured by FMD but not CIMT [27]. Nonetheless this study comprised only 80 patients with bronchiectasis and 80 controls. The study also did not stratify patients with bronchiectasis according to disease severity.

A large-scale study to compare CIMT in patients with bronchiectasis and healthy subjects is warranted to assess the risks of subclinical atherosclerosis, taking into account disease severity. In view of this knowledge gap, we conducted this study with the objective being assessing burden of subclinical atherosclerosis as measured by CIMT among patients with bronchiectasis of different severity, as well as comparing CIMT between patients with bronchiectasis and healthy controls.

## Materials and methods

A prospective study was conducted at the University Department of Medicine, Queen Mary Hospital (QMH). The Divisions of Respiratory Medicine and Neurology at Queen Mary Hospital are tertiary referral centers for the territory as well as major receiving units for patients with various respiratory diseases (including bronchiectasis) and neurological diseases in the Hong Kong West Cluster. Subjects above the age of 18 years old with a confirmed diagnosis of bronchiectasis based on high-resolution computed tomography (HRCT) scan were included. Those with co-existent systemic inflammatory diseases (Rheumatological diseases, inflammatory bowel diseases and other autoimmune diseases) and respiratory co-morbidities (asthma, chronic obstructive pulmonary disease and interstitial lung diseases) were excluded. HRCT and lung function tests were performed within 12 months prior to recruitment to the study. Severity of bronchiectasis was defined according to the FACED [forced expiratory volume in 1 s (FEV_1_), age, chronic colonization, extension, and dyspnea score] score, with mild, moderate and severe bronchiectasis defined as a FACED score of 0–2, 3–4, and 5–7 points respectively [28]. Subjects with bronchiectasis were recruited from 1st October to 31st December 2021 from the bronchiectasis clinic of QMH. Controls without bronchiectasis, as assessed by symptom questionnaires and checking of electronic patient records of the Hospital Authority of Hong Kong, were recruited between July 2018 and June 2020 from the community by open advertisements.

As gender and smoking status are the important factors that could contribute to differences in CIMT, while they are also significantly different in the two groups, they are chosen to be factors for propensity score matching. Control patients were individually matched by gender and smoking status using propensity score matching with a 1:2 matching ratio and caliper of 0.2. Covariates that were not well-matched were adjusted in multivariate analysis.

Vascular ultrasound examination of carotid intima-medial thickness (CIMT) was performed by a standard B-mode ultrasound examination with a 7.5 MHz linear array transducer and a high-resolution ultrasound system in accordance with the Mannheim Carotid Intima-Media Thickness and Plaque Consensus [29–31]. In patients with bronchiectasis, ultrasound examination was performed by a single experienced operator (Kwok Wang Chun) using General Electric LOGIQ e. In controls, scans were performed by one of four operators (Matthew SS Hsu, Kkts Pijarnvanit, Carman Nga-Man Cheung, Yick Hin Chow) using a Samsung Ultrasound RS80A. All subjects were examined in a supine position. Ultrasound scans of the right and left common carotid artery in three different projections (anterior, lateral, and posterior) were performed. CIMT was determined by measuring the distance between the lumen-intima and media-adventitia border at a 10 mm straight arterial segment near the bulb at the far wall of both common carotid arteries. CIMT was calculated using automated IMT software. CIMT was measured at each projection (anterior, lateral, and posterior) from each side giving six measurements for each patient and the mean value calculated.

Seventeen randomly selected controls underwent CIMT re-measurement, performed with General Electric LOGIQ e by the same operator who assessed bronchiectasis patients to identify any inter-machine variability.

Automated IMT function was used to measure CIMT in both bronchiectasis patients and control, which has been demonstrated to have high accuracy and reproducibility [32–34]. Automated IMT measurement allows automated contour detection of lumen-intima and media-adventitia vessel walls and calculation of IMT quality index. The advantage of having automated IMT measurement is that it avoided some of the problems from manual measurement of CIMT, including being user-dependent, measurement not fully standardized, subjective, time-consuming and prone to errors [35].

The primary outcome was the difference in CIMT between patients with bronchiectasis and controls, and among patients with bronchiectasis of different severity as determined by FACED score. The study was approved by the Institutional Review Board of the University of Hong Kong and Hospital Authority Hong Kong West Cluster (UW19-624).

### Statistical analysis

The demographic and clinical data are presented as actual frequency, mean ± standard deviation (SD) or median (interquartile range, IQR) as appropriate. Categorical variables were compared using χ2 test. For continuous variables, two-group comparisons were performed using unpaired t test or Mann-Whitney U test as appropriate. One-way ANOVA was employed to compare multi-group continuous variables. Multivariate linear regression analyses were performed to adjust for cofounders that included age, gender, smoking history, body mass index (BMI), cardiovascular risk factors and history of cardiovascular diseases. Statistical significance was determined at the level of *p* = 0.05. All statistical analyses were performed using R V.4.2.2 (R Foundation for Statistical Computing) statistical software.

## Results

A total of 155 Chinese patients with non-CF bronchiectasis managed at QMH and 512 controls were included. The baseline characteristics of the patients with bronchiectasis and the controls are summarized in Table 1.

**Table 1:** Baseline demographic and clinical characteristics of all subjects

	Controls (*n* = 512)	Bronchiectasis patients (*n* = 155)	*P*-value
Age (years), mean ± SD	54.5 ± 6.6	69.0 ± 11.4	< 0.01*
Male (%)	255 (45.5%)	54 (34.8%)	< 0.01*
Smoking status (%)			0.18
Current smoker	38 (7.4%)	5 (3.2%)	
Former smoker	71 (13.9%)	21 (13.6%)	
Non-smoker	403 (78.7%)	129 (83.2%)	
Body mass index (kg/m^2^), mean ± SD	24.0 ± 4.6	22.2 ± 3.9	< 0.01*
CIMT (mm), mean ± SD	0.58 ± 0.10	0.64 ± 0.11	< 0.01*
Hypertension (%)	91 (17.8%)	47 (30.3%)	< 0.01*
Diabetes mellitus (%)	19 (3.7%)	17 (11.0%)	< 0.01*
Hyperlipidemia (%)	66 (12.9%)	28 (18.1%)	0.11
Ischemic heart disease (%)	1 (0.2%)	11 (7.1%)	< 0.01*
Ischemic stroke/Transient ischemic attack (%)	0 (0%)	3 (1.9%)	0.01*
FEV_1_ (L), mean ± SD	-	1.69 ± 0.66	-
FEV_1_ (% predicted), mean ± SD	-	86.3 ± 24.5	-
FVC (L), mean ± SD	-	2.50 ± 0.85	-
FVC (% predicted), mean ± SD	-	95.7 ± 20.6	-
FEV_1_/FVC Ratio (%), mean ± SD	-	68.2 ± 12.5	-
Extent of involvement ≥ 3 lobes (%)	-	60 (38.7%)	-
*Pseudomonas aeruginosa* colonization (%)	-	49 (31.6%)	-
Colonization by other organisms (%)		16 (10.3%)	-
Exacerbation(s) in the past 1 year (%)	-	33 (21.3%)	-
Number of exacerbation(s) in the past 1 year (%)			
1	-	17 (11.0%)	-
2	-	7 (4.5%)	-
3	-	4 (2.6%)	-
4	-	3 (1.9%)	-
5	-	2 (1.3%)	-
Hospitalized exacerbation(s) in the past 1 year (%)	-	12 (7.7%)	-
Number of hospitalized exacerbation(s) in the past 1 year (%)			
1	-	10 (6.5%)	-
2	-	2 (1.3%)	-
Baseline FACED score, Median (IQR)	-	3 (2–4)	-
Baseline BSI score, mean ± SD	-	5.5 ± 3.2	-
Baseline blood neutrophil count (x cells/µL), mean ± SD	-	4.10 ± 1.95	-
Baseline blood lymphocyte (x cells/µL), mean ± SD	-	1.89 ± 0.71	-
Etiology of bronchiectasis			
Post-tuberculosis	-	32 (20.6%)	-
Non-tuberculosis mycobacteria infection	-	14 (9.0%)	-
Post-irradiation	-	3 (1.9%)	-
Diffuse pan-bronchiolitis	-	1 (0.6%)	-
Post-haemopoietic stem cell transplantation	-	3 (1.9%)	-
Primary ciliary dyskinesia	-	3 (1.9%)	-
Idiopathic	-	99 (63.9%)	-
Medication for bronchiectasis (%)			
Macrolide	-	25 (16.1%)	-
Inhaled corticosteroid	-	57 (36.8%)	-
LABA		35 (22.6%)	-
Theophylline	-	7 (4.5%)	-

SD = standard deviation; CIMT = carotid intima-medial thickness; mL = milliliter; * = statistically significant; FEV_1_ = forced expiratory volume in one second; FVC = forced vital capacity, BSI = Bronchiectasis severity index, LABA = Long-acting beta-agonist

### Correlation of CIMT among controls using different ultrasound machines

Both the General Electric LOGIQ e and Samsung Ultrasound RS80A were used to measure CIMT in 17 controls. All scans were performed by the same investigator. Measurements of CIMT by both machines had a high Pearson Correlation Coefficient of 0.856 (95% confidence interval [CI] = 0.627–0.949, *p* < 0.001).

### Interrater variability of CIMT among controls

Ultrasound measurement of CIMT in controls was performed by four operators. The interrater variability was determined by repeat ultrasound CIMT measurement in 11 controls by different investigators. The intraclass correlation coefficient was 0.977.

### Whole cohort with 155 patients with bronchiectasis and 512 controls

#### CIMT in patients with bronchiectasis and controls (whole cohort, *n* = 667)

Patients with bronchiectasis had significantly increased CIMT compared with controls (0.64 ± 0.11 mm vs. 0.58 ± 0.10 mm respectively, *p* < 0.001). The association was statistically significant after adjusting for age, gender, BMI, smoking status, any cardiovascular risk factors and any history of cardiovascular diseases (*p* = 0.020) (Supplementary Table S1).

#### *CIMT in patients with bronchiectasis and controls without a history of cardiovascular disease or cardiovascular risk factors (Bronchiectasis*, *n* = 92; control, *n* = 384)

The mean CIMT, as compared by unpaired t test, among patients with bronchiectasis and controls was 0.61 ± 0.10 mm and 0.57 ± 0.10 mm respectively (*p* < 0.001). The association was statistically significant after adjusting for age, gender, BMI, smoking status, any cardiovascular risk factors, and any history of cardiovascular disease in multivariate linear regression (*p* = 0.007) (Supplementary Table S1).

#### *CIMT in patients with bronchiectasis of different severity and controls (Mild-to-moderate bronchiectasis*,*n* = 126; Severe bronchiectasis,*n* = 29; control,*n* = 512)

**Fig. 1:**
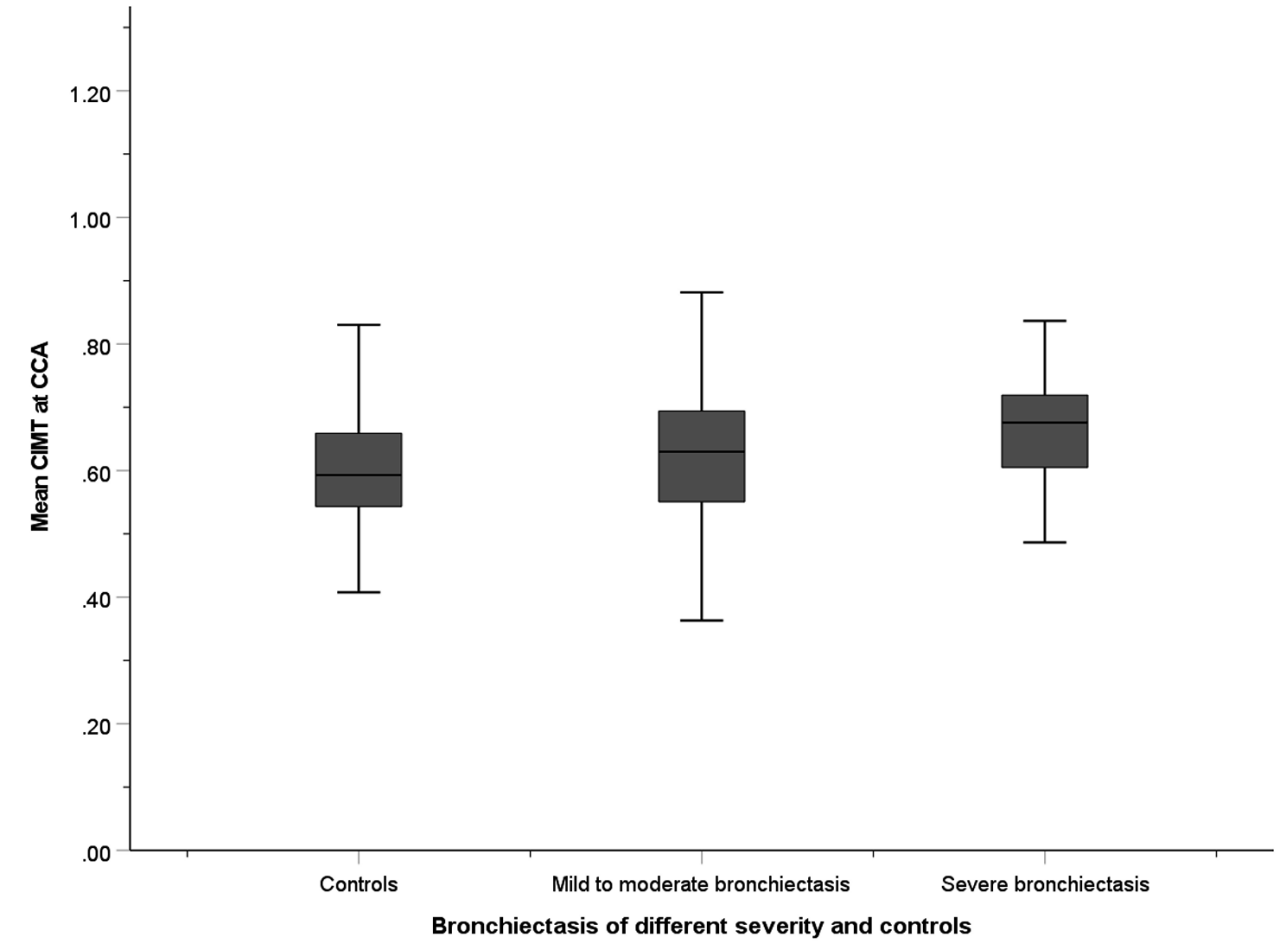
Mean CIMT among all patients with mild-to-moderate bronchiectasis, severe bronchiectasis, and controls in the whole cohort. CIMT are compared by one-way ANOVA. CIMT: Carotid intima-media thickness

The CIMT, compared by one-way ANOVA, was 0.58 ± 0.10 mm, 0.63 ± 0.11 mm and 0.66 ± 0.08 mm among controls, patients with mild-to-moderate bronchiectasis and patients with severe bronchiectasis respectively (Fig. 1). The association was statistically significant after adjusting for age, gender, BMI, smoking status, any cardiovascular risk factors, and any history of cardiovascular disease in multivariate linear regression (*p* = 0.021) (Supplementary Table S1).

#### *CIMT in patients with bronchiectasis of different severity and controls with no history of cardiovascular disease or cardiovascular risk factors (Mild-to-moderate bronchiectasis*,*n* = 76; Severe bronchiectasis,*n* = 16; control,*n* = 384)

**Fig. 2:**
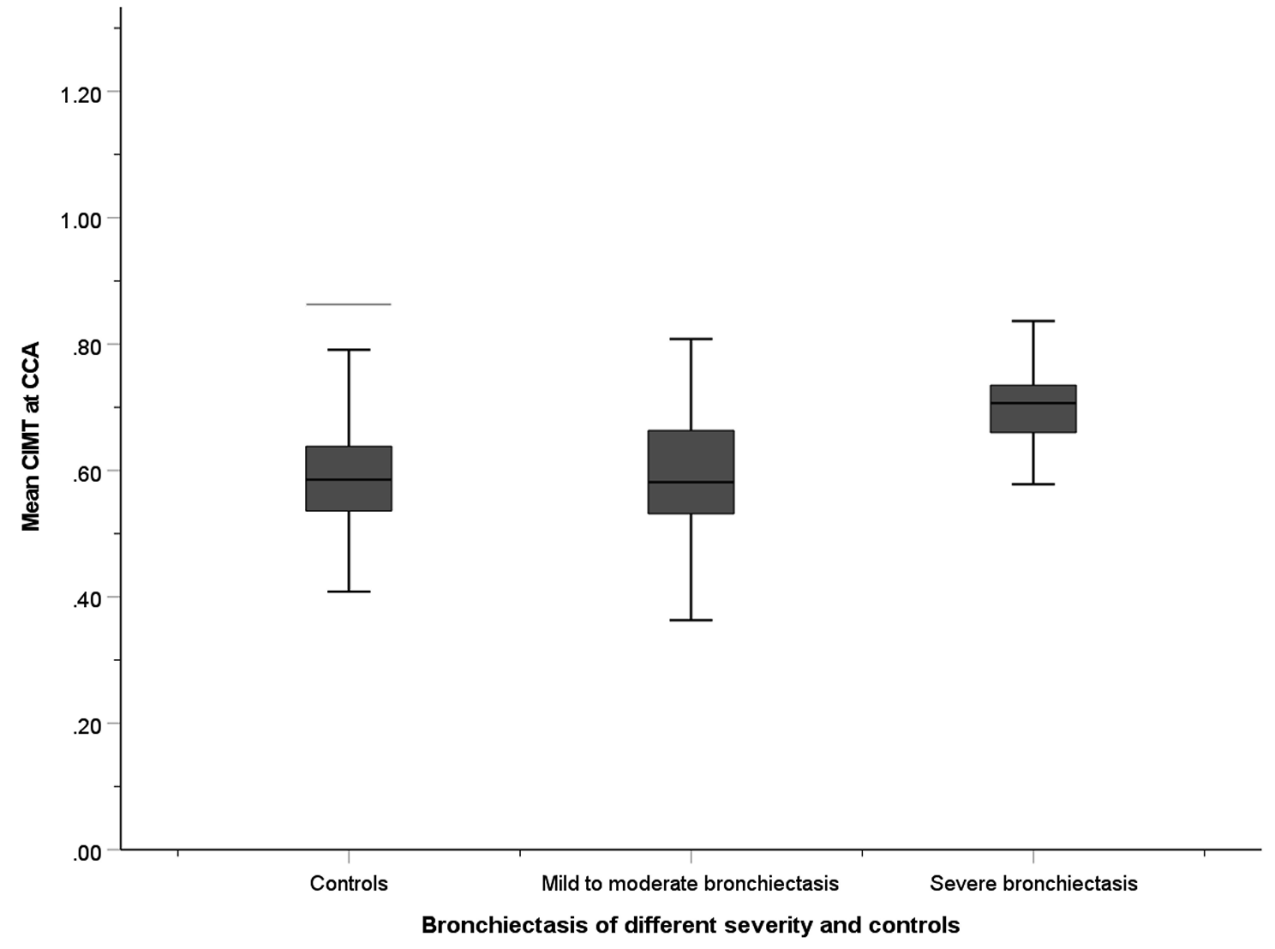
Mean CIMT among patients with mild-to-moderate bronchiectasis, severe bronchiectasis, and controls with no cardiovascular risk factors or history of cardiovascular diseases in the whole cohort. CIMT are compared by one-way ANOVA. CIMT: Carotid intima-media thickness

The CIMT, compared by one-way ANOVA, was 0.57 ± 0.10 mm, 0.60 ± 0.10 mm and 0.69 ± 0.06 mm among controls, patients with mild-to-moderate bronchiectasis and patients with severe bronchiectasis (Fig. 2). The association was statistically significant after adjusting for age, gender, BMI, smoking status, any cardiovascular risk factor, and any history of cardiovascular disease in multivariate linear regression (*p* < 0.001) (Supplementary Table S1).

### Matched cohort (*n* = 465)

There were 155 subjects with bronchiectasis and 310 controls in the propensity score matched cohort (Table 2).

**Table 2:** Baseline demographic and clinical characteristics of subjects in matched cohort

-	Controls (*n* = 310)	Bronchiectasis patients (*n* = 155)	*P*-value
Age (years), mean ± SD	55.0 ± 6.6	69.0 ± 11.4	< 0.01*
Male (%)	108 (34.8%)	54 (34.8%)	1.00
Smoking status (%)			0.275
Current smoker	20 (6.5%)	5 (3.2%)	
Former smoker	34 (11.0%)	21 (13.6%)	
Non-smoker	256 (82.6%)	129 (83.2%)	
Body mass index (kg/m^2^), mean ± SD	23.7 ± 5.2	22.2 ± 3.9	< 0.01*
CIMT (mm), mean ± SD	0.58 ± 0.10	0.64 ± 0.11	< 0.01*
Hypertension (%)	50 (16.1%)	47 (30.3%)	< 0.01*
Diabetes mellitus (%)	7 (2.3%)	17 (11.0%)	< 0.01*
Hyperlipidemia (%)	35 (11.3%)	28 (18.1%)	0.06
Ischemic heart disease (%)	0 (0%)	11 (7.1%)	< 0.01*
Ischemic stroke/Transient ischemic attack (%)	0 (0%)	3 (1.9%)	0.01*
FEV_1_ (L), mean ± SD	-	1.69 ± 0.66	-
FEV_1_ (% predicted), mean ± SD	-	86.3 ± 24.5	-
FVC (L), mean ± SD	-	2.50 ± 0.85	-
FVC (% predicted), mean ± SD	-	95.7 ± 20.6	-
FEV_1_/FVC Ratio (%), mean ± SD	-	68.2 ± 12.5	-
Extent of involvement ≥ 3 lobes (%)	-	60 (38.7%)	-
*Pseudomonas aeruginosa* colonization (%)	-	49 (31.6%)	-
Colonization by other organisms (%)		16 (10.3%)	-
Exacerbation(s) in the past 1 year (%)	-	33 (21.3%)	-
Number of exacerbation(s) in the past 1 year (%)			
1	-	17 (11.0%)	-
2	-	7 (4.5%)	-
3	-	4 (2.6%)	-
4	-	3 (1.9%)	-
5	-	2 (1.3%)	-
Hospitalized exacerbation(s) in the past 1 year (%)	-	12 (7.7%)	-
Number of hospitalized exacerbation(s) in the past 1 year (%)			
1	-	10 (6.5%)	-
2	-	2 (1.3%)	-
Baseline FACED score, Median (IQR)	-	3 (2–4)	-
Baseline BSI score, mean ± SD	-	5.5 ± 3.2	-
Severity of bronchiectasis by FACED score			
Severe	-	29 (18.7%)	-
Moderate	-	60 (38.7%)	-
Mild	-	66 (42.6%)	-
Baseline blood neutrophil count (x cells/µL), mean ± SD	-	4.10 ± 1.95	-
Baseline blood lymphocyte (x cells/µL), mean ± SD	-	1.89 ± 0.71	-
Etiology of bronchiectasis			
Post-tuberculosis	-	32 (20.6%)	-
Non-tuberculosis mycobacteria infection	-	14 (9.0%)	-
Post-irradiation	-	3 (1.9%)	-
Diffuse pan-bronchiolitis	-	1 (0.6%)	-
Post-haemopoietic stem cell transplantation	-	3 (1.9%)	-
Primary ciliary dyskinesia	-	3 (1.9%)	-
Idiopathic	-	99 (63.9%)	-
Medication for bronchiectasis (%)			
Macrolide	-	25 (16.1%)	-
Inhaled corticosteroid	-	57 (36.8%)	-
LABA		35 (22.6%)	-
Theophylline	-	7 (4.5%)	-

SD = standard deviation; CIMT = carotid intima-medial thickness; mL = milliliter; * = statistically significant; FEV_1_ = forced expiratory volume in one second; FVC = forced vital capacity

#### CIMT in patients with bronchiectasis and controls

Patients with bronchiectasis had significantly increased CIMT compared with controls (0.64 ± 0.11 mm and 0.58 ± 0.10 mm respectively, *p* < 0.001), as compared by unpaired t-test. The association was statistically significant after adjusting for age, gender, BMI, smoking status, any cardiovascular risk factor, and any history of cardiovascular disease in multivariate linear regression (*p* = 0.020) (Supplementary Table S2).

#### *CIMT in patients with bronchiectasis and controls with no history of cardiovascular disease or cardiovascular risk factors (Bronchiectasis*, *n* = 92; control,*n* = 235)

The mean CIMT, compared by unpaired t-test, among patients with bronchiectasis and controls was 0.61 ± 0.10 mm and 0.57 ± 0.09 mm respectively (*p* < 0.001). The association was statistically significant after adjusting for age, gender, BMI, smoking status, any cardiovascular risk factor and any history of cardiovascular disease in multivariate linear regression (*p* = 0.017) (Supplementary Table S2).

#### *CIMT in patients with bronchiectasis of different severity and controls (Mild-to-moderate bronchiectasis*,*n* = 126; Severe bronchiectasis,*n* = 29; control,*n* = 310)

The mean CIMT, as compared by one-way ANOVA, was 0.58 ± 0.10 mm, 0.63 ± 0.11 mm and 0.66 ± 0.08 mm among controls, patients with mild-to-moderate and severe bronchiectasis respectively. The association was statistically significant after adjusting for age, gender, BMI, smoking status, any cardiovascular risk factor and any history of cardiovascular disease in multivariate linear regression (*p* = 0.004) (Supplementary Table S2).

#### *CIMT in patients with bronchiectasis of different severity and controls with no history of cardiovascular disease or cardiovascular risk factors (Mild-to-moderate bronchiectasis*,*n* = 76; Severe bronchiectasis,*n* = 16; control,*n* = 235)

The mean CIMT was 0.56 ± 0.09 mm, 0.60 ± 0.10 mm and 0.69 ± 0.06 mm respectively among controls, patients with mild-to-moderate and patients with severe bronchiectasis. The association was statistically significant after adjusting for age, gender, BMI, smoking status, any cardiovascular risk factor, and any history of cardiovascular disease (*p* < 0.001) (Supplementary Table S2).

### Subgroup analysis – age > 60

There were 126 subjects with bronchiectasis and 102 controls above the age of 60 years in the propensity score matched cohort. Age is reported to be associated with CIMT [36], with age 60 years a commonly used cut-off to define age group in CIMT studies [37–40]. The results in this subgroup show consistent results as in the primary analysis. The findings of subgroup analysis were summarized in Supplementary Table S3 and S4.

## Discussion

To the best of our knowledge, this is the first report of a significant association of severe bronchiectasis with CIMT, but not mild-to-moderate bronchiectasis. The findings were consistent among patients with or without cardiovascular risk factors or cardiovascular disease. Among patients with severe bronchiectasis, defined by a FACED score 5 or above, CIMT was increased compared with controls.

There is growing evidence of an association of adverse cardiovascular outcomes with bronchiectasis. A possible link between the two is chronic inflammation, a hallmark of bronchiectasis. Nonetheless no previous study has suggested that CIMT is increased in patients with bronchiectasis. A small-scale case control study identified only a difference in FMD, not CIMT. This may have been due to the small sample size of only 80 patients with bronchiectasis and 80 controls. Our study has a larger sample size so overcomes the potential problem of the previous case control study that lacked statistical power to detect CIMT differences. In addition, previous study did not take account of disease severity. In our study, bronchiectasis as a whole group (a group with varying severity) may not account for the differences in CIMT. Nonetheless based on analysis according to disease severity, we determined that severe bronchiectasis, not mild-to-moderate, was associated with increased CIMT. We postulate that there is an interplay of various factors that underlie the association of bronchiectasis and CIMT. This finding is consistent with the proposal that CIMT, as a marker of subclinical atherosclerosis, is related to the degree of inflammation. The more inflamed the airway is, the thicker the CIMT. This finding is supported by studies in other inflammatory diseases. In rheumatoid arthritis, features of more severe disease, including extra-articular manifestations, erosions, high inflammatory parameters, and long disease duration, were associated with greater CIMT [41]. A similar phenomenon has been observed in systemic lupus erythematosus and psoriasis [42, 43]. This may explain why CIMT is increased in patients with severe bronchiectasis but not mild-to-moderate cases. We postulate that as an inflammatory disease mainly affecting the respiratory tract, the severity of bronchiectasis is closely related to the degree of systemic inflammation and hence CIMT. For patients with mild-to-moderate bronchiectasis, the degree of systemic inflammation is low and CIMT is not increased.

CIMT was also being assessed in other respiratory diseases before. In chronic obstructive pulmonary disease (COPD), 32% of patients with mild COPD and 36% with moderate to severe COPD had increased CIMT, compared with 23% in patients without COPD [44]. CIMT was also found to be positively correlated with exacerbation rate in past year and negatively correlated with FEV_1_ among patients with COPD, which suggested that CIMT is related to COPD severity [45]. CIMT was also reported to be increased among patients with asthma, [46] which may have differences in different phenotypes. In Atherosclerosis Risk in Communities (ARIC) study, CIMT was increased among women with adult-onset asthma but not childhood-onset asthma [47]. This could be related to the severity of asthma as adult-onset asthma which is well reported to be associated with higher morbidity and mortality [48, 49]. In cystic fibrosis, CIMT was increased among patients who are pancreatic insufficient but not those who are pancreatic sufficient [50].

In patients with severe bronchiectasis, as defined by FACED score, they are more likely to have *Pseudomonas colonization*, worse lung function, and more symptomatic as measured by mMRC dyspnoea scale. They are also older with more lobes of the lungs being involved. By having *Pseudomonas colonization*, this will lead to chronic low-grade airway inflammation. The chronic inflammation can contribute to an increase in CIMT as in other inflammatory diseases. Worse lung function by FEV_1_, higher mMRC dyspnoea scale and more extensive disease could reflect the airway damage from chronic airway inflammation, which is linked to the increase in CIMT. The chronic inflammatory state in these patients with severe bronchiectasis could eventually lead to subclinical atherosclerosis with an increase in CIMT. The constellation of all these factors, as reflected in FACED score, ultimately translate into increase in CIMT, mediated through heightened inflammatory state in these patients.

Our findings provide a pathophysiological basis for the observed association of bronchiectasis with cardiovascular diseases. Although systemic inflammatory diseases have been reported to be associated with cardiovascular diseases, the evidence for bronchiectasis is weaker, especially from a pathogenic aspect. The only evidence for subclinical atherosclerosis derived from a small-scale study of FMD [27] and brachial-ankle pulse wave velocity (baPWV), a measure of arterial stiffness [51]. Our study is the first to show evidence of an association of bronchiectasis with increased CIMT in a severity-dependent manner. Together with the previous reports on FMD and baPWV, [27, 51] these findings provide a stronger pathogenic basis to support the reported association of bronchiectasis with cardiovascular diseases. In previous literature, bronchiectasis has been shown to be associated the adverse cardiovascular outcomes. But as a heterogenous disease, the exact pathophysiological mechanism is not established. This could be attributed by smoking, advanced age and co-existing diseases. Our study provides data to suggest that the association of bronchiectasis and cardiovascular diseases could be contributed by bronchiectasis itself, if severe enough. As bronchiectasis is associated with cardiovascular diseases, we believe that using CIMT as a non-invasive tool can help to identify at risk population, especially patients with severe bronchiectasis and underlying cardiovascular risk factors. The findings from our study also call for the screening and monitoring of cardiovascular health in patients with bronchiectasis, especially if it is a severe one. CIMT is one of the options while other clinical parameters such as blood pressure, glucose and lipid level shall also be assessed. These patients may need better control of bronchiectasis and cardiovascular risk factors, for example with smoking cessation, lowering of lipid level and achieve optimal blood pressure control, to prevent future cardiovascular events. Although bronchiectasis cannot be reversed, yet, it can be controlled with various medications like macrolide and inhaled antibiotics, which may help to prevent bronchiectasis exacerbation and probable subsequent cardiovascular events after bronchiectasis exacerbation.

In bronchiectasis, one of the goals of pharmacotherapy is immunomodulation, such as by macrolide and inhaled corticosteroid (ICS). Whether these treatments can have effect on CIMT and subsequent cardiovascular events worth further research, Macrolide has been shown to reduce the frequency of exacerbations and improve quality of life in bronchiectasis [52]. It acts by suppressing bacterial infection and reducing airway inflammation. ICS works in selected group of patients stratified by the blood eosinophil count [53]. ICS exerts the effect through altering macrophage gene expression, decreasing interferon (IFN)-γ expression and upregulating chemokine production [54]. The clinical benefits of these treatments in bronchiectasis are mainly on the respiratory outcomes such as bronchiectasis exacerbation. It is interesting to know if they can offer cardiovascular protective effect through dampening down the degree of airway inflammation and later reduction in CIMT, which is a marker of subclinical atherosclerosis.

There are some limitations to our study. First, it involved only a single centre with all the patients included being Chinese. This could have potential implication of generalizability of the findings in different ethnic groups, in which the etiology of bronchiectasis could be different. Also, as a single centre study, the sample size is relatively small, especially for patients with severe bronchiectasis. Nonetheless as a tertiary medical centre, the respiratory unit receives referrals from all other health care facilities across the territory. Patients diagnosed with bronchiectasis are managed in a designated bronchiectasis clinic at our centre. And the association of CIMT and severity of bronchiectasis can be demonstrated in this study with statistical significance. A large-scale multi-centre study involving different ethnic group will be worthwhile to conduct to assess if the same phenomenon is observed across different ethnic groups. Second, CIMT was measured using different ultrasound machines for patients with bronchiectasis and controls. Although there may be inter-machine variability for measurement of CIMT, we validated the findings in 17 controls and demonstrated a good correlation for measurements obtained by the two ultrasound machines. In propensity score matching, ideally, all factors that are significantly different should be matched. However, given a relatively small sample size, especially in the severe bronchiectasis group. Matching all the factors that are significantly different such as age and co-morbidities would lead to loss of some of the patients with severe bronchiectasis which cannot be matched. As such, we matched only the gender and smoking status, while adjusting the other factors that are significantly different but not matched are included in multi-variate analysis as confounder. Another limitation is that lung function and blood test results available. They were identified by symptom questionnaire and electronic health record. This may potentially include some patients with minute lung function abnormality or any systemic disorder. Yet, they have been screened for any underlying respiratory diseases by questionnaire and their electronic health record. As they are free of respiratory symptoms without physician diagnosed respiratory diseases, the possibility of erroneously recruiting healthy controls with major respiratory or systemic diseases that affect the CIMT is very low. Also, in the bronchiectasis group, the mean baseline FEV_1_ was 86.3 ± 24.5%, with 104 patients with baseline FEV_1_ > 70%. The baseline neutrophil and lymphocyte count in the bronchiectasis group is also relatively normal. The impact from lung function and abnormal blood count on the result is considered to be minute, if there is any.

## Conclusions

CIMT was significantly increased in patients with severe bronchiectasis compared with controls without bronchiectasis, but not among patients with mild-to-moderate bronchiectasis, which suggested the subclinical atherosclerosis to be more prevalent among patients with severe bronchiectasis.

### Electronic supplementary material

Below is the link to the electronic supplementary material.

Supplementary Material 1

Supplementary Material 2: Appendix 1 Questionnaire for symptom screening in healthy control

## Data Availability

Dataset supporting the conclusion of this article is included within this article and no additional data will be provided. Research data is not shared.

## Abbreviations

AMI ankle: Brachial index
baPWV brachial: Ankle pulse wave velocity
BMI: Body mass index
CHD: Coronary heart disease
CIMT: Carotid intima-media thickness
CRP: C-reactive protein
FMD: Flow-mediated dilatation
HRCT: High-resolution computed tomography
IQR: Interquartile range
SD: Standard deviation

## Declarations

### Clinical Trial Details

Not Applicable. We do not have a Clinical Trial Number as it is not a clinical trial. We only have IRB approval number.
