# Supplementary material for: Severe bronchiectasis is associated with increased carotid intima-media thickness
Source: BMC Cardiovasc Disord. 2024 Aug 28;24:457. doi: 10.1186/s12872-024-04129-x (PMC11350994; doi:10.1186/s12872-024-04129-x)
Supplement: Supplementary file 1 — Supplementary Material 1 [file 12872_2024_4129_MOESM1_ESM.docx]

**Supplementary table 1 Multivariate linear regression analysis results in unmatched cohort**

|  | **Unstandardized Beta coefficient** | **Standard error** | **p-value** |
| --- | --- | --- | --- |
| ***Bronchiectasis vs control*** | 0.091 | 0.020* | 0.020* |
| ***Patients with bronchiectasis and controls without a history of cardiovascular disease or cardiovascular risk factors*** | 0.118 | 0.007* | 0.007* |
| ***Patients with bronchiectasis of different severity and controls*** | 0.090 | 0.021* | 0.021* |
| ***Patients with bronchiectasis of different severity and controls with no history of cardiovascular disease or cardiovascular risk factors*** | 0.179 | < 0.001* | < 0.001* |

**Supplementary table 2 Multivariate linear regression analysis results in matched cohort**

|  | **Unstandardized Beta coefficient** | **Standard error** | **p-value** |
| --- | --- | --- | --- |
| ***Bronchiectasis vs control*** | 0.024 | 0.010 | 0.020* |
| ***Patients with bronchiectasis and controls without a history of cardiovascular disease or cardiovascular risk factors*** | 0.024 | 0.011 | 0.017* |
| ***Patients with bronchiectasis of different severity and controls*** | 0.014 | 0.006 | 0.004* |
| ***Patients with bronchiectasis of different severity and controls with no history of cardiovascular disease or cardiovascular risk factors*** | 0.016 | 0.001 | < 0.001* |

*: Statistically significant with p < 0.05

**Supplementary table 3** **Mean CIMT among patients with bronchiectasis and controls in subgroup age > 60**

|  | **Mean CIMT (mm)** | **p-value**  **(Unpaired t-test)** | **p-value**  **(Multivariate linear regression) ^#^** |
| --- | --- | --- | --- |
| ***Unmatched cohort*** | | |  |
| Bronchiectasis  (n = 126) | 0.66 ± 0.09 | <0.001* | 0.087 |
| Control  (n = 102) | 0.62 ± 0.10 |  |  |
| ***Subjects with no history of cardiovascular disease or cardiovascular risk factors*** | | | |
| Bronchiectasis  (n = 68) | 0.65 ± 0.08 | < 0.001* | 0.004* |
| Control  (n = 71) | 0.60 ± 0.07 |  |  |

CIMT: Carotid intima-media thickness

#: Adjusted for age, gender, BMI, smoking status, any cardiovascular risk factor or any history of cardiovascular disease

*: Statistically significant with p < 0.05

**Supplementary table 4 Mean CIMT among patients with bronchiectasis of different severity and controls in subgroup age > 60**

|  | **Mean CIMT (mm)** | **p-value**  **(One-way ANOVA)** | **p-value**  **(Multivariate linear regression) ^#^** |
| --- | --- | --- | --- |
| ***Unmatched cohort*** | | | |
| Mild to moderate bronchiectasis  (n = 97) | 0.63 ± 0.10 | 0.011* | 0.490 |
| Severe bronchiectasis  (n = 29) | 0.66 ± 0.08 |  |  |
| Control  (n = 102) | 0.62 ± 0.10 |  |  |
| ***Subjects with no history of cardiovascular disease or cardiovascular risk factors*** | | | |
| Mild to moderate bronchiectasis  (n = 52) | 0.64 ± 0.08 | < 0.001* | < 0.001* |
| Severe bronchiectasis  (n = 16) | 0.69 ± 0.06 |  |  |
| Control  (n = 71) | 0.60 ± 0.07 |  |  |

CIMT: Carotid intima-media thickness

#: Adjusted for age, gender, BMI, smoking status, any cardiovascular risk factor or any history of cardiovascular disease

*: Statistically significant with p < 0.05
