# Supplementary material for: Severe bronchiectasis is associated with increased carotid intima-media thickness
Source: BMC Cardiovasc Disord. 2024 Aug 28;24:457. doi: 10.1186/s12872-024-04129-x (PMC11350994; doi:10.1186/s12872-024-04129-x)
Supplement: Supplementary file 2 — Supplementary Material 2: Appendix 1 Questionnaire for symptom screening in healthy control [file 12872_2024_4129_MOESM2_ESM.docx]

Any New Diagnosis for the Following Disease

對以下疾病的任何新診斷

- Any Hospitalization for any Reason after Recruitment 加入研究後的曾因任何原因需要住院治療
- No
- Yes
- Rifaximin / Probiotics 最近使用利福昔明或益生菌
- No
- Yes

Reason for Hospitalization

- Gastro-intestinal surgery or Endoscopy 腸胃手術或腸胃鏡檢查
- No
- Yes
- Gastro-intestinal surgery or Endoscopy 腸胃手術或腸胃鏡檢查 (choice=Endoscopy 腸胃鏡檢查)
- Type of GI surgery / Endoscopy Results
- Gastro-intestinal Disease 胃腸病症 (choice=No)
- Gastro-intestinal Disease 胃腸病症 (choice=Peptic Ulcer Disease 胃及十二指腸潰瘍)
- Gastro-intestinal Disease 胃腸病症 (choice=Gastritis / Duodenitis 胃炎/十二指腸炎)
- Gastro-intestinal Disease 胃腸病症 (choice=Upper GI Bleed 腸上道出血)
- Gastro-intestinal Disease 胃腸病症 (choice=Lower GI Bleed 腸下道出血)
- Gastro-intestinal Disease 胃腸病症 (choice=Persistent Dyspepsia 持續性消化不良)
- Gastro-intestinal Disease 胃腸病症 (choice=Gastric polyps 胃瘜肉)
- Gastro-intestinal Disease 胃腸病症 (choice=Colonic polyps 大腸瘜肉)
- Gastro-intestinal Disease 胃腸病症 (choice=Inflammatory bowel syndrome 炎性腸病)
- Gastro-intestinal Disease 胃腸病症 (choice=Irritable bowel syndrome 腸易激綜合徵)
- Gastro-intestinal Disease 胃腸病症 (choice=Helicobacter pylori infection 幽門螺旋桿菌感染)
- Stroke / TIA 中風/短暫性腦缺血中風
- No
- Yes
- Ischaemic Heart Disease (CAD/IHD/Angina) 缺血性心臟病
- No
- Yes
- Hypertension 高血壓
- No
- Yes
- Diabetes 糖尿病
- No
- Yes
- DM Complication 糖尿併發症 (choice=Retinopathy 糖尿眼)
- DM Complication 糖尿併發症 (choice=Nephropathy 糖尿腎病)
- DM Complication 糖尿併發症 (choice=Neuropathy 糖尿神經病變)
- DM Complication 糖尿併發症 (choice=None of the above 以上皆不是)
- Hyperlipidaemia 高血脂
- No
- Yes
- Atrial Fibrillation 心房顫動
- No
- Yes
- Venous Thrombosis 靜脈栓塞
- No
- Yes
- Peripheral Vessel Disease 外周血管疾病史
- No
- Yes
- Obstructive Sleep Apnea 阻塞性睡眠呼吸暂停
- No
- Yes
- Positive Airway Pressure Use 持續性正壓呼吸器
- No
- Yes
- Epilepsy 癲癇
- No
- Yes
- Other: Please specify 其他 請明確說明
- Drug Allergy 藥物過敏
- No
- Yes
- Drug Allergy Please specify:
- Hepatitis 肝炎
- If yes,
- Hepatitis Type (choice=Hepatitis A)
- Hepatitis Type (choice=Hepatitis B)
- Hepatitis Type (choice=Hepatitis C)
- Hepatitis Type (choice=Hepatitis E)
- Hepatitis Type (choice=Other)
- Chronic Kidney Disease or known renal impairment 慢性腎病或已知的腎功能受損
- Renal Replacement Therapy 腎替代治療法 (choice=No)
- If yes,
- Renal Replacement Therapy 腎替代治療法 (choice=CAPD 腹膜透析)
- Renal Replacement Therapy 腎替代治療法 (choice=HD 血液透析)
- Renal Replacement Therapy 腎替代治療法 (choice=Transplant 腎移植)
- Chronic Obstructive Pulmonary Disease 慢性阻塞性肺疾病
- No
- Yes
- Asthma 哮喘
- No
- Yes
- Psychiatric disorder 心理疾病
- No
- If yes:
- Psychiatric disorder 心理疾病 (choice=Depression 抑郁症)
- Psychiatric disorder 心理疾病 (choice=Anxiety disorder 焦慮症)
- Psychiatric disorder 心理疾病 (choice=Schizophenia 思覺失調症)
- Psychiatric disorder 心理疾病 (choice=Bipolar affective disorder 精神分裂症)
- Other: Please specify 其他 請明確說明
- Delivery mode at birth? 出生時的分娩方式
- Breast feed at birth? 出生時母乳喂養
- Breast feed duration 母乳喂養持續時間
- Menopause ? 停經一年以上 (has not had a period for one year)
- If Menopause/Hysterectomy done, When?
- Do you have any follow up at HA? Or been visit any HA facility? 你有醫院管理局的跟進嗎？ 或者去過醫院管理局的任何設施？
